# The repeatability of neuromuscular activation strategies recorded in recreationally active individuals during cycling

**DOI:** 10.1007/s00421-022-04899-2

**Published:** 2022-02-15

**Authors:** Hannah R. Cutler, Emma Hodson-Tole

**Affiliations:** 1 Musculoskeletal Science and Sports Medicine Research Centre, Dpt. Life Sciences, Manchester Metropolitan University, Manchester, UK; 2 Manchester Metropolitan University Institute of Sport, Manchester, UK

**Keywords:** Surface electromyography, Muscle coordination, Cycling, Motor redundancy, Repeatability

## Abstract

**Purpose:**

The human neuro-motor system can select different intermuscular coordination patterns to complete any given task, such as pedalling a bicycle. This study assessed whether intermuscular coordination patterns are used consistently across visit days and cadence conditions in recreationally active individuals.

**Methods:**

Seven participants completed a cycling exercise protocol across 2 days, consisting of pedalling at 150 Watts at cadences of 60, 80 and 100 rpm. Whilst cycling, surface electromyography was continuously recorded from ten leg muscles. For each participant, muscle coordination patterns were established using principal component (PC) analysis and the amount that each pattern was used was quantified by the PC loading scores. A sample entropy derived measure of the persistence of the loading scores across consecutive pedal cycles, entropic half-life (EnHL), was calculated. The median loading scores and EnHLs of the first three PCs were then compared across cadence conditions and visit days.

**Results:**

No significant differences were found in the median loading scores across cadence conditions or visits, nor were there any significant differences in the EnHLs across visits. However, the EnHLs were significantly longer when participants were cycling at 60 rpm compared to 100 rpm.

**Conclusion:**

These findings are based on a small sample size, but do suggest that, within individual participants, a consistent neuromuscular control strategy is used during cycling on different days. However, the underlying structure of muscle coordination is more persistent when pedalling at slower cadences with large differences between individuals.

## Introduction

When humans pedal a bicycle, coordination of multiple lower limb muscles must occur to generate and apply the required reaction forces to the pedal. The amount that each muscle is recruited can vary across consecutive pedal cycles. This may reflect motor redundancy, the suggestion that the body accommodates more muscles than mechanical degrees of freedom at the joints (Bernshteĭn 1967). Accordingly, there are a number of solutions available to the nervous system to solve a given motor task (Muller and Sternad 2009), providing humans with the adaptability to meet task-specific requirements and optimise performance. To investigate the motor recruitment patterns used during cycling, surface electromyography (sEMG) can be employed to record the temporospatial summation of motor unit action potentials. Through analysing the peaks of sEMG waveforms, the contribution of individual muscles to the pedal cycle has been characterised (Houtz and Fischer 1959).

Surface EMG is often used in clinical and sports research to evaluate changes in muscle recruitment and coordination parameters over time (Vigotsky et al. 2018). Several studies have examined the repeatability of sEMG bursts within individuals cycling under the same conditions across multiple days. These studies have demonstrated high reproducibility of the magnitude components of the signals (Laplaud et al. 2006; MacDonald et al. 2008; Travis et al. 2011; Bini et al. 2017), with one finding high reproducibility of the temporal components (Jobson et al. 2013). These findings suggest that the same muscle activation patterns are consistently used during cycling, indicating good potential for these measures to be used for monitoring changes in clinical and sports training settings. Nevertheless, through examining individual muscles separately, minimal information is obtained regarding the coordinative strategies that are key in limiting performance (Blake and Wakeling 2015).

To explore intermuscular coordination, principal component analysis (PCA) can be applied to sEMG signals recorded from multiple muscles. PCA reduces the dimensionality of large EMG datasets, capturing the most relevant features of the signals in a small set of principal components (PCs). Each PC constitutes an orthogonal mode of variation present within the input data matrix and has frequently been used to represent a muscle coordination pattern (Chung et al. 2012; Blake and Wakeling 2015; Enders et al. 2015; Hodson-Tole et al. 2020; Turpin et al. 2011; Qi et al. 2021). Each component is characterised by loading scores which determine how much it (the component) contributes to the total signal at any timepoint. Six PCs are shown to sufficiently explain ≥ 95% of the variance in sEMG datasets recorded from ten leg muscles, of experienced cyclists, during cycling (Wakeling and Horn 2009; Hodson-Tole et al. 2020). In trained cyclists, coordination patterns show robust consistency across a range of torque–velocity combinations (Hug et al. 2011), and are therefore considered to reflect some degree of neural control. However, consistency of coordination patterns in experienced cyclists may reflect their training and/or practice history, and thus may not be found in recreationally active individuals who have more limited training experiences. The repeatability of coordination patterns has not, however, been investigated in recreationally active individuals. Given populations of clinical patients are unlikely to be dominated by skilled cyclists it is important to identify whether recreationally active individuals show consistency in coordination patterns to facilitate evaluation of interventions, such as those aimed at rehabilitating motor function in stroke survivors.

In addition to investigating the instantaneous patterns of muscle recruitment and coordination, recent studies have evaluated time dependency in cycle-to-cycle fluctuations in recruitment and coordination (Enders et al. 2015; Hodson-Tole and Wakeling, 2017; Wakeling and Hodson-Tole, 2018) to reveal features of the underlying motor control processes. One method used for such investigation is a sample entropy (SampEn)-based analysis that quantifies the rate at which the structure (temporal organization of variability) of a signal decays, using a measure termed entropic half-life (EnHL) (Zandiyeh and Von Tscharner 2013). Analysis of sEMG signals recorded during cycling have revealed structure exists in muscle coordination patterns, and changes in response to alterations in cadence (Enders et al. 2015; Wakeling and Hodson-Tole 2018; Hodson-Tole et al. 2020) and load (Wakeling and Hodson-Tole 2018; Hodson-Tole et al. 2020). This suggests alterations in the number of solutions available or used by the nervous system to meet the task demand. Repeatability in the structure of coordination patterns for cycling between different measurement sessions has, however, never been investigated, meaning that the consistency in the underlying motor control processes is currently unknown.

Therefore, the purpose of this study was to use PCA and EnHL to identify whether neuromuscular activation strategies are consistently used during cycling in recreationally active individuals. The main muscle coordination patterns used by an individual will be identified using PCA, and to assess repeatability, the PC loading scores will be compared across cadence conditions and visit days. EnHLs will be used to assess time dependency in coordination across consecutive pedal cycles, and values for each participant will be compared across cadence conditions and visits. We hypothesise that consistent intermuscular coordination patterns will be used during cycling, across visits and cadence conditions in recreationally active individuals.

## Methods

Seven male (*n* = 4) and female (*n* = 3) healthy volunteers (mean ± SD: age, 24 ± 3.9 years; mass 71.9 ± 10.7 kg; height 175.7 ± 9 cm) provided written informed consent to participate in the study, which was approved by Manchester Metropolitan University Ethics Committee and conducted in accordance with the Declaration of Helsinki. All participants were physically active, typically completing moderate to vigorous intensity exercise 3 to 4 days per week. Exclusion criteria included anyone with lower extremity musculoskeletal injuries or cardiovascular health problems in the prior six months, and anyone who cycled more than 20 h per week, on average. Participants attended two testing sessions, at least a week apart, consisting of a 22-min cycling exercise protocol, whilst sEMG recordings were made.

## Protocol

**Fig. 1:**
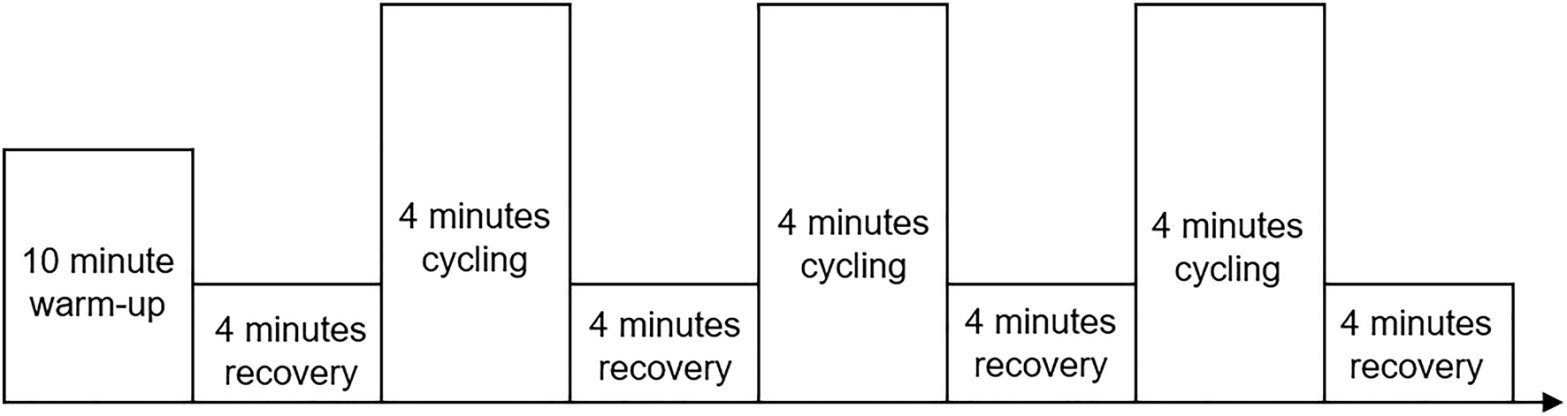
Experimental design: a randomised design consisting of 3 × 4-min bouts of cycling at 60, 80 or 100 rpm, whilst at a power output of 150 Watts, including active recovery periods where participants cycled at a freely chosen cadence

The cycling protocol commenced with a 10-min warm up, cycling at a freely chosen cadence; followed by three four-minute bouts of pedalling at 60, 80 and 100 rpm with a recovery period between each bout consisting of active rest where participants cycled at a freely chosen cadence (Fig. 1). All cycling exercises were completed on a calibrated braked electromagnetic cycle ergometer (Schoberer Rad Meßtechnik, Germany), equipped with a torque sensing crank. The heights of the saddle and the handlebars were adjusted so that participants were seated in a traditional cycling posture with a knee flexion angle of approximately five degrees when the pedal was at the bottom dead centre. Measurements of the saddle height and handlebars were recorded during the first visit to ensure that participants were seated in the same position during the subsequent visit.

During each trial, cadence conditions were randomly selected, and participants were asked to maintain the required cadence as close as possible to the target, via visual and verbal feedback. Whilst the participants settled into pedalling, the load was adjusted to match the required cadence so that each participant was cycling at a constant power output of 150 Watts, at each cadence. This power output was selected as it represented a sustainable load that recreationally active individuals could maintain for at least four minutes. Once participants were cycling at the necessary cadence and power output, sEMG data recording and a four-minute timer were started. 

## sEMG data acquisition

Muscle activity was continuously recorded during the cycling protocol from ten lower limb muscles of the right leg, specifically the: gluteus maximus (GMax), vastus lateralis (VL), rectus femoris (RF), vastus medialis (VM), semitendinosus (ST), biceps femoris (BF), gastrocnemius lateralis (GL), gastrocnemius medialis (GM), soleus (SOL) and tibialis anterior (TA). After shaving, exfoliating, and cleaning the skin, bipolar Ag/AgCl sEMG electrodes (10 mm diameter, 21 mm interelectrode distance; Trigno Wireless EMG System, Delsys) were placed on the centre of the muscle bellies, according to the recommendations of SENIAM guidelines (Hermens et al. 2000), ensuring that the electrodes were positioned away from the innervation zone to avoid any extraneous noise.

The researchers were experienced with the procedure of electrode placement and care was taken to consistently identify anatomical landmarks and electrode position in accordance with (Hermens et al. 2000) and these are detailed here in Table 1. During the first visit, limb segment lengths and relative electrode position measurements were recorded to facilitate consistent electrode placement during the second visit. While the exact electrode location was unlikely to be obtained between visits (and we did not attempt to quantify differences) our approach will reflect the differences likely to occur in many repeated measures design sEMG studies, for example, to assess effects of an exercise intervention. The sEMG signals were sampled (2000 Hz) through a USB data acquisition device (X-Series-USB 6341, National Instruments, Austin, Texas, USA) and saved on a laptop using custom-written code (LabView, Version 9.0, National Instruments). Cadence was recorded concurrently with the sEMG signals through the same USB data acquisition device and code, using a Hall-effect sensor positioned at the top dead centre of the crank cycle.

**Table 1:** Electrode placement according to the SENIAM Guidelines (Hermens et al. 2000)

Muscle	Location	Orientation
Tibialis anterior	1/3 on the line between the tip of the fibula and tip of the medial malleolus	In the direction of the line between tip of fibula and tip of medial malleolus
Gluteus maximus	50% on the line between the sacral vertebrae and the greater trochanter	In the direction of the line from the posterior superior iliac spine to the middle of the posterior part of the thigh
Vastus lateralis	2/3 on the line from the anterior spina iliaca superior to the lateral side of the patella	In the direction of the muscle fibres
Rectus femoris	50% on the line from the anterior spina iliaca superior to the superior part of the patella	In the direction of the line from the anterior spina iliaca superior to the superior part of the patella
Vastus medialis	80% on the line between the anterior spina iliaca superior and the joint space in front of the anterior border of the medial ligament	Almost perpendicular to the line between the anterior spina iliaca superior and the joint space in front of the anterior border of the medial ligament
Semitendinosus	50% on the line between the ischial tuberosity and the medial epicondyle of the tibia	In the direction of the line between the ischial tuberosity and the medial epicondyle of the tibia
Biceps femoris	50% on the line between the ischial tuberosity and the lateral epicondyle of the tibia	In the direction of the line between the ischial tuberosity and the lateral epicondyle of the tibia
Gastrocnemius lateralis	1/3 on the line between the head of the fibula and the heel	In the direction of the line between the head of the fibula and the heel
Gastrocnemius medialis	On the most prominent bulge of the muscle	In the direction of the leg
Soleus	2/3 of the line between the medial condylis of the femur to the medial malleolus	In the direction of the line between medial condylis to the medial malleolus

## sEMG data processing

The sEMG data were analysed to identify the main features of muscle activity patterns and coordination within each visit. As individuals are known to have unique patterns of muscle activation (Hug et al. 2019) we quantified muscle activation and coordination within each participant. To do this, the sEMG signals were decomposed into time–frequency space using an EMG specific wavelet analysis (von Tscharner 2000), with a filter bank of 11 wavelets (0 ≤ *k* ≤ 10, frequency bandwidth 11 to 432 Hz). Frequency spectra were screened to ensure signals were not dominated by low frequency noise e.g. due to poor skin–electrode interface and movement artefact (De Luca et al. 2010; Huigen et al. 2002). The total intensity was calculated as the sum of intensities from wavelets *k* = 1 to *k* = 10, providing a measure of the power of the signals at each timepoint reducing any contribution of low frequency noise associated with motion artefacts (De Luca et al. 2010). For each participant, the total intensity for each muscle was normalised using the mean maximum amplitude of all cycles for each muscle per visit, so data were normalized within visit. Pedal cycle durations were defined as the time between consecutive peaks in the pedal switch data. Total intensity data were split into individual pedal cycles, defined from the pedal switch. To ensure consistency in the number of pedal cycles analysed per trial (important for later SampEn calculations), the first 232 pedal cycles from each trial were taken forward for analysis. The total number of pedal cycles analysed was 9744 (7 participants × 2 visits × 3 conditions × 232 pedal cycles).

## Principal component analysis

PCA was used to quantify the variance in each participant’s sEMG dataset that, given the biological basis of sEMG signals, were considered predominantly representative of muscle coordination patterns. As the PCA was completed on each individual participant’s data, within participant variance was quantified and may therefore represent different features of recorded sEMG signals across participants. For each condition, the coordination patterns per pedal cycle were created from the normalised EMG intensities for all 10 muscles. Subsequently, a *p* × *N* matrix **A **was constructed where *p* represented the number of muscles (10) and *N* conveyed the number of pedal cycles (232 × 2 visits × 3 conditions). The PCs were determined by Eigen analysis of covariance matrix **B** with no prior subtraction of the mean (Wakeling and Rozitis 2004). The PC weightings were given by the eigenvector **ξ **of covariance matrix **B**, with negative values allowing for representation of possible signal reductions reflecting inhibitory control within the muscle excitation signals. The PC loading scores were determined from the eigenvalues. In PCA, typically the mean is subtracted at the beginning of the analysis, meaning that the eigenvectors explain a set of components that maximise the variance from the mean. However, as the mean was not subtracted in this analysis, the eigenvalues explain a set of components which maximise the variability of the dataset (Wakeling and Rozitis 2004). This means that PC1 represents the mean and the amount of signal variability that the mean represents is quantified. The PC loading score represents the amount each PC, or coordination pattern, was used for each pedal cycle analysed and the overall coordination pattern for any given cycle can be approximated through a linear combination of the PCs and their respective loading scores. Therefore, the overall coordination patterns used by each participant across cadence conditions and visit days were reconstructed as a linear combination of the first three PC weightings scaled by the corresponding mean loading score for the condition and visit. All data analysis was completed using custom-written code (Mathematica 12.1.1.0, Wolfram Research Inc.).

## Sample entropy calculation

**Fig. 2:**
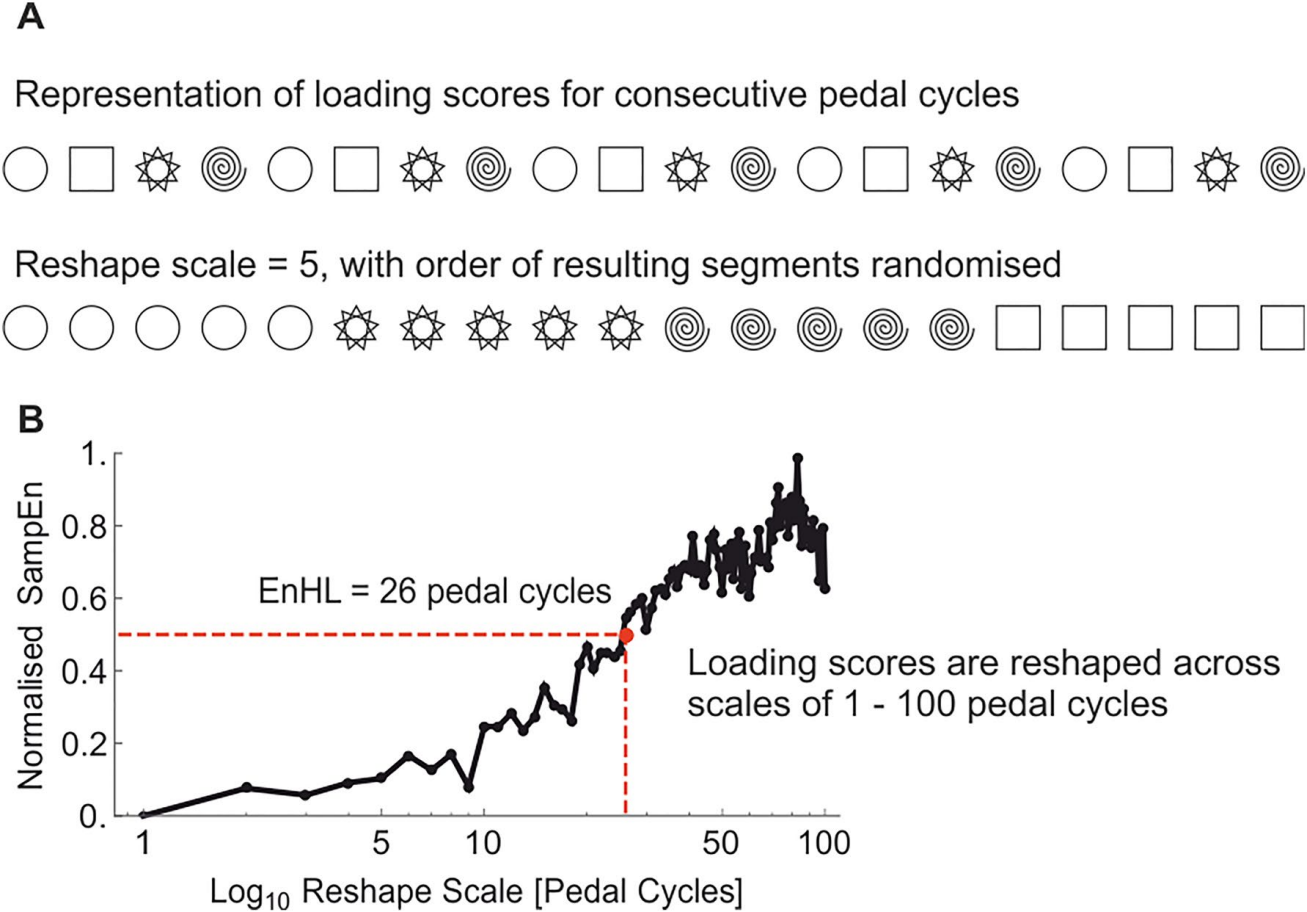
**A** Schematic representation of the reshape scale process, illustrating a reshape scale of 5 such that every 5th loading score is selected and the resulting data segments concatenated in random order (to avoid introduction of correlations at longer time scales). **B** Example of normalized SampEn values resulting from the reshape scale process. SampEn = 0.5 represents the transition from structured to random variation in the analysed time series, here indicating an EnHL = 26 pedal cycles

To quantify the EnHLs, a reshape scale method spanning 1 to 100 pedal cycles was used to reshape the PC loading score time series (Zandiyeh and von Tscharner, 2013), and a freely available software package (Goldberger et al. 2000) was used to calculate SampEn for each signal (Fig. 2). The reshape scale evaluates the timescale at which data points (pedal cycle loading scores) are affiliated with each other by rearranging the signal over several scales. SampEn then quantifies the amount of similarity in the signal*,* based on the probability that two patterns of *m* consecutive data points with tolerance of *r*, will remain similar when an additional data point is added (*m* + 1). Values of *m* = 2 and *r* = 0.2 were used. The SampEn values were normalised using the equation defined by Raffalt and Yentes (2018),

$${\text{Normalised}}\,{\text{SampEn}} = \frac{{{\text{SampEn}}_{{{\text{RS}}}} - {\text{SampEn}}_{{{\text{OR}}}} }}{{{\text{SampEn}}_{m = 0} - {\text{SampEn}}_{OR} }}$$

where SampEn_RS_ is the SampEn of the reshape time series, SampEn_OR_ is the SampEn of the original time series and SampEn_m = 0_ is the SampEn of the randomised time series. Once normalised, SampEn values were plotted as a function of the reshape scales. The EnHL was determined as the time scale at which SampEn = 0.5, indicating the transition time at which the reshaped time series changes from predictable to random (Fig. 2).

## Statistical analysis 

Continuous variables were tested for normality using both the Kolmogorov–Smirnov and Shapiro-Wilks test (SPSS, Version 26, IBM), with concurring results from each test for all variables. For normally distributed variables, the rANOVA was used (SPSS, Version 26). Meanwhile, for data that were not normally distributed, a non-parametric equivalent of the rANOVA, the F1-LD-F1 model, was used (Brunner et al. 2002). This model was implemented using the ‘nparLD’ package (Noguchi et al. 2012) in R.4.0.4 (R Core Team 2018). To check that participants adhered to the desired cadence in each trial, the rANOVA was used to compare the average number of pedal cycles for each participant across the visit days, with cadence as the dependent variable and participants as the independent variable. To assess repeatability, the rANOVA was also used to compare the median PC loading scores and EnHLs across visit days and cadence conditions. Additionally, the rANOVA was used to compare the EnHLs across PC vectors to identify whether they needed to be considered independently. For these analyses, PC loading scores and EnHLs were considered dependent variables and cadence, visit day and participant were defined as independent variables. Pairwise comparisons of the EnHLs across cadence conditions were completed, using the ‘nparLD’ package. Results were reported as median ± IQR for non-parametric variables and mean ± SD for parametric variables. All results were deemed significant at *p* ≤ 0.05.

## Results

### Adherence to the experimental protocol

**Fig. 3:**
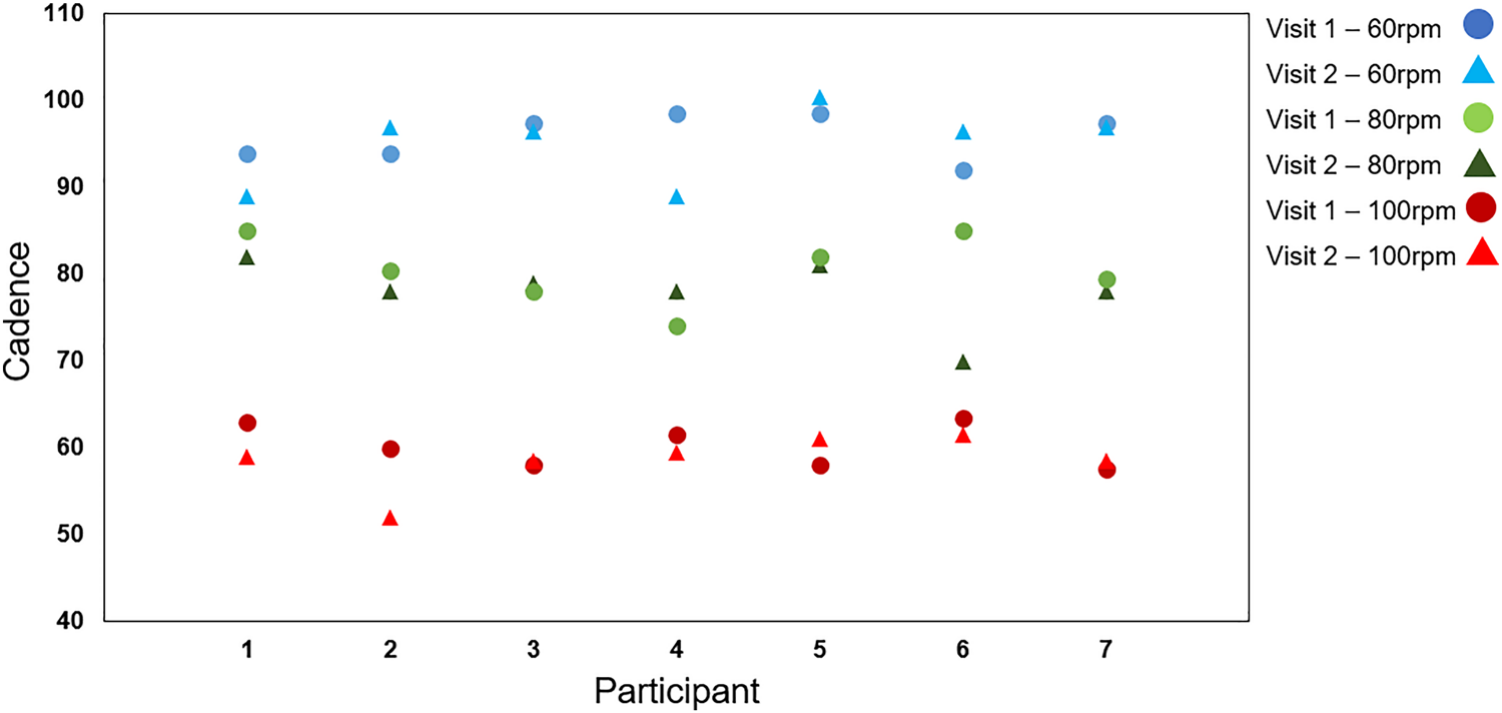
Dot plots of the average cadence (revolutions per minute) for each participant*.* The points for each cadence condition: 60, 80 and 100 rpm are shown in blue, green, and red, respectively. Each circle represents the cadence maintained on visit 1 and each triangle represents the cadence maintained on visit 2

On average, cadence was 2.1 rpm lower than the target velocities required and varied with a standard deviation of 2.7 rpm. Across the three cadence conditions of 60, 80 and 100 rpm, the mean cadences were 59.7 ± 0.7 rpm, 78.4 ± 0.9 rpm, and 95.1 ± 0.9 rpm, respectively. No significant differences were found in the mean cadence across the two visits for any of the participants when cycling at 60, 80 or 100 rpm (*F* = 0.248, *p* = 0.863) (Fig. 3), suggesting that intersession repeatability was observed. Cycling load was adjusted to match the cadence chosen during each condition so it can be assumed that power output was maintained at approximately 150 Watts by all participants, as required by experimental design.

### Principal component analysis

**Fig. 4:**
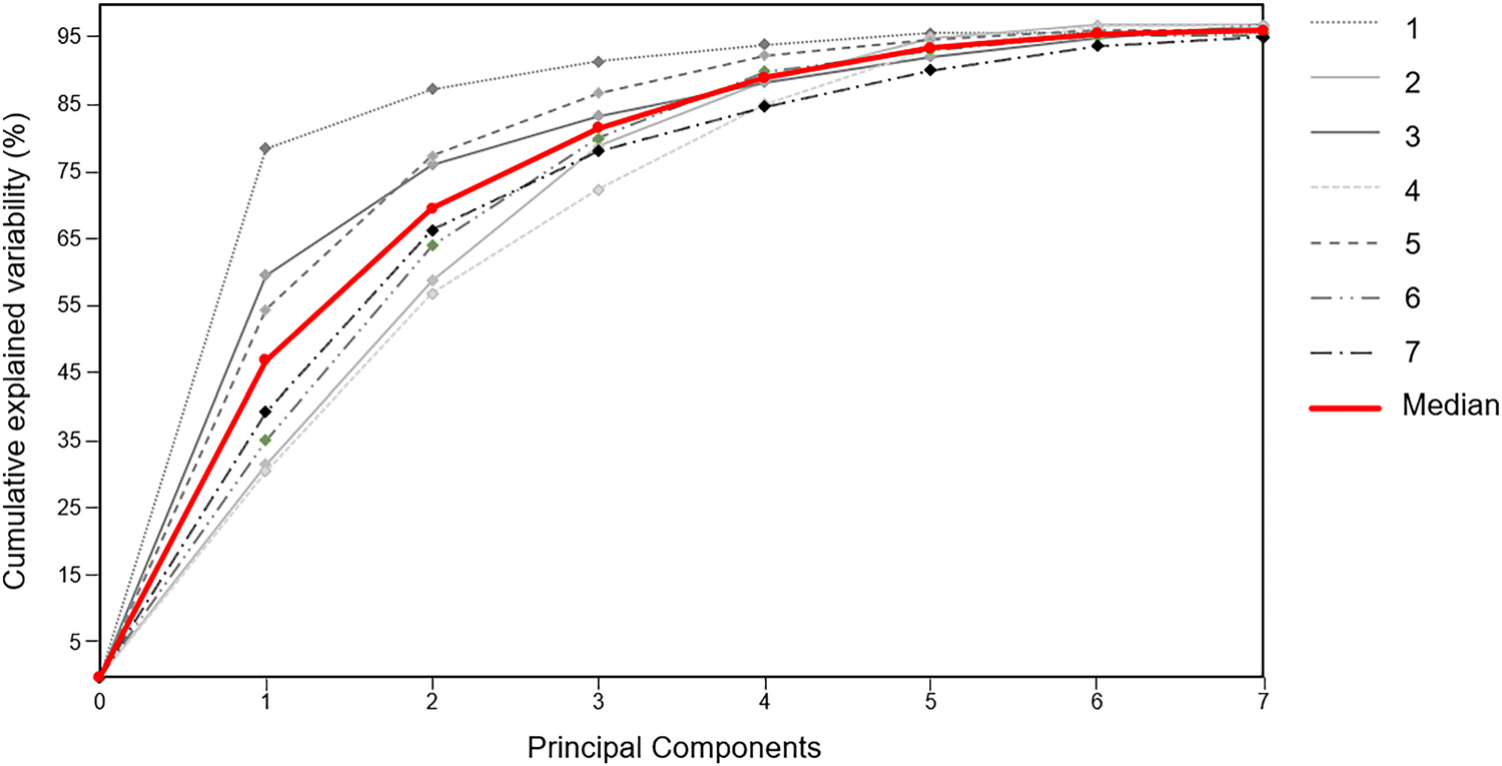
The principal components (*horizontal axis*) required by the participants (*N* = 7*)* to explain up to 95% of the variability in the EMG dataset (*vertical axis)*. Each line represents the cumulative percentage of variability explained by the PCs for each individual participant. The median of these data is shown in red

From the PCA five to seven PCs explained ≥ 95% of the variability in the sEMG dataset recorded across all cycling sessions in each participant. The cumulative percentage of variability explained by each component is shown in Fig. 4. On average, PC1 explained 39.3% of the variability, ranging from 30.6% to 78.5% across the participants. Meanwhile, PC2 and PC3 on average explained 26.4% ± 20.1% and 11.7% ± 15.8% of the total variability, respectively.

**Fig. 5:**
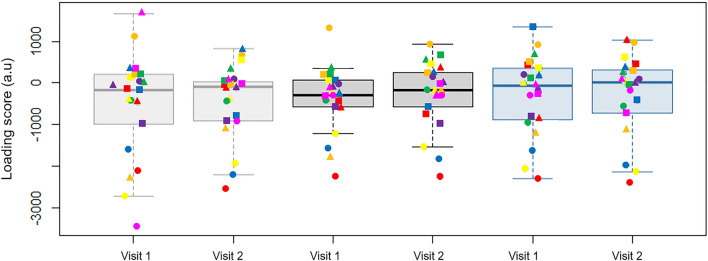
Boxplots for the median principal component loading scores of the first three PCs for each cadence condition and visit day. The box plots for each cadence condition: 60, 80 and 100 rpm are shown in grey, black, and blue, respectively. The data points for PC 1, 2 and 3 are represented as circles, triangles, and squares, respectively. Each colour represents the same participant across each cadence condition and visit day (*N* = 7). For each condition, the boxplot for visit one is on the left and visit two is on the right

To assess repeatability, the loading scores of the first three PCs were compared across cadence conditions and visit days (Fig. 5). Overall, the rANOVA found no significant differences in the median loading scores of the participants on visit one (*F* = 0.481, *p* = 0.616) or visit two (*F* = 0.345, *p* = 0.703), indicating that the main PCs were used to the same extent when pedalling at each cadence on the two visits. Equally, across the two visits, the rANOVA found no significant differences in the median loading scores when participants were cycling at 60 (*F* = 0.017, *p* = 0.894), 80 (*F* = 2.251, *p* = 0.133) or 100 rpm (*F* = 3.593, *p* = 0.058), suggesting that the average amount the PCs were used at each cadence was also consistent on different visit days.

### Reconstructed muscle coordination patterns

**Fig. 6:**
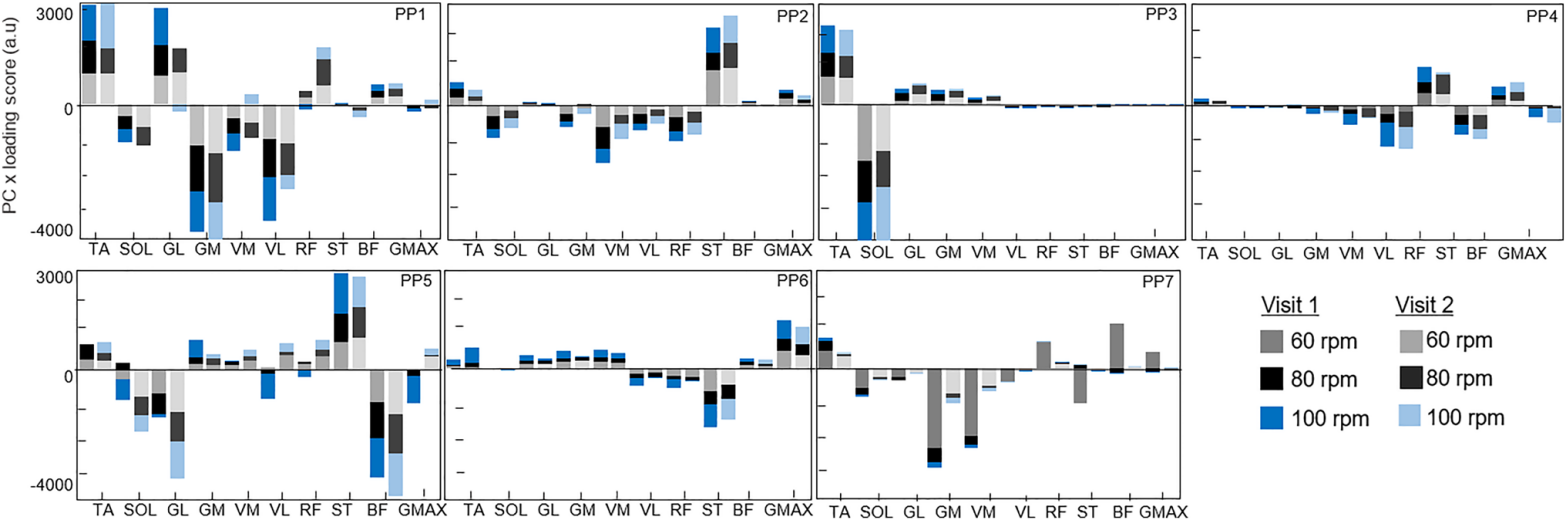
The main muscle recruitment patterns used by each participant when cycling at 60, 80 and 100 rpm, across the two visits. Each bar represents the amount that each muscle was used during each condition on visit one (left) and visit two (right). The cadence conditions of 60, 80 and 100 rpm are shown as grey, black, and blue bars, respectively. The bars are a product of the first three PCs and their vector loadings (*vertical axis*) for each muscle (*horizontal axis)*. *TA* tibialis anterior, *SOL* soleus, *GL* gastrocnemius lateralis, *GM* gastrocnemius medialis, *VM* vastus medialis, *VL* vastus lateralis, *RF* rectus femoris, *ST* semitendinosus, *BF* biceps femoris, *GMAX* gluteus maximus

The overall coordination patterns used by each participant across cadence conditions and visit days were reconstructed as a linear combination of the first three PC weightings scaled by their corresponding loadings scores (Fig. 6). These components cumulatively explained approximately 79.9% ± 19.1% of the sEMG dataset recorded across all conditions and visits. The reconstructed coordination patterns demonstrate the relative levels of activity between the muscles, so the positive and negative bars indicate that some muscles are active when others are inhibited. On visual inspection of the reconstructions, similar muscles can be seen grouped together. The quadricep muscles: VM, VL and RF appear to work closely with each other, and with the GM, GL, and SOL. Meanwhile the ST, BF, and TA, appear to work antagonistically to these muscles. Nevertheless, across the participants, there are large interindividual variations in the amount that each of these muscles are used. These variations are particularly evident in the bi-articulate muscles, such as the GL and ST. However, less variation can be seen in mono-articulate muscles such as the VL, TA and SOL.

### Entropic half-life of the PCs

The EnHLs were calculated for the first three PCs for all participants across each cadence condition and visit day. Each EnHL represents the number of pedal cycles over which the persistence of the main muscle recruitment patterns decayed over. Greater EnHL indicates increased persistence of signal regularity, so one pedal cycle has influence over a greater number of future cycles. Lower values indicate less persistence, with fewer future pedal cycles influenced. Across all conditions, the EnHLs obtained were invariably above 1, ranging from 2 to 52 pedal cycles with a median of 4.7 ± 31.3 pedal cycles. This indicates that across all cycling sessions there was some degree of persistence in the use of the main PCs.

Overall, there was significant main effect of the PC vector on the EnHLs (*F* = 3.109, *p* = 0.039), indicating that the degree of persistence in the signals differed between muscle coordination patterns. Specifically, the median EnHLs across PCs 1, 2 and 3 were 4 ± 50, 2 ± 25 and 4 ± 25 pedal cycles, respectively. This indicates that PC1 and PC3 demonstrated the most amount of signal persistence, whilst PC2 was characterised by the least amount of signal persistence. Therefore, when considering the effects of visit day and cadence on the EnHLs, each PC was considered independently.

**Fig. 7:**
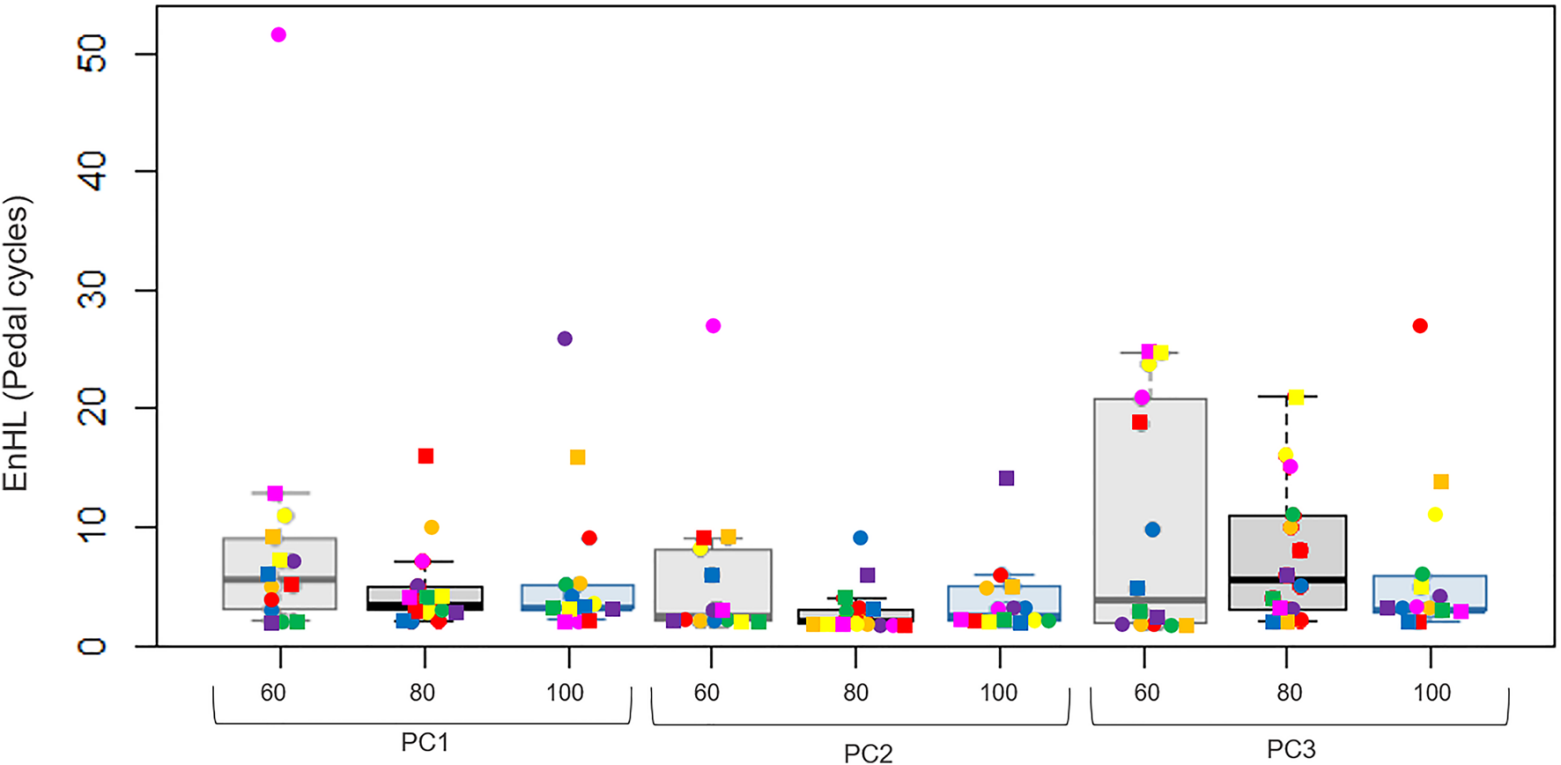
Boxplots of the EnHLs (*vertical axis)* across cadence conditions for each PC vector (*horizontal axis)*. The cadence conditions 60, 80 and 100 rpm are shown in grey, black and blue, respectively. Each data point represents the EnHL of a participant (*N* = 7) and each colour represents the same participant across each condition and visit day. Visit one and visit two are shown as circles and squares, respectively

To assess repeatability, the EnHLs for each PC vector were compared across the two visit days when cycling at each cadence (Fig. 7). The rANOVA of the overall EnHLs found no significant effects of visit day for PC1 (*F* = 1.179, *p* = 0.305), PC2 (*F* = 0.379, *p* = 0.537) or PC3 (*F* = 1.853*, p* = 0.158*)*. Furthermore, the rANOVA found no significant effects of cadence on the EnHLs for PC3 (*F* = 0.105, *p* = 0.851). However, a significant effect of cadence was found in PC1 (*F* = 3.846, *p* = 0.049) and PC2 (*F* = 5.109, *p* = 0.032). Subsequent pairwise comparisons of the individual cadence conditions highlighted a significant increase in the EnHLs when participants were cycling at 60 rpm, compared to 100 rpm in both PC1 (*p* = 0.014) and PC2 (*p* = 0.049). In detail, the median EnHLs at 60, 80 and 100 rpm were 4.5 ± 50, 3 ± 19 and 3 ± 25 pedal cycles, respectively. This indicates that the EnHLs persisted over more pedal cycles at 60 rpm compared to the other cadences.

## Discussion

This study explored the intersession repeatability of the intermuscular coordination patterns used during cycling by recreationally active individuals. Through PCA we were able to identify the principal components of recorded sEMG, which we interpret as predominantly representing the participant’s coordination patterns, and use EnHL to quantify the underlying structure within them. This approach expanded on studies which have examined the repeatability of global EMG waveforms of individual muscles, and importantly studied inexperienced, recreationally active, cyclists. Overall, five to seven PCs were used by the participants. The contributing muscle coordination patterns can be visualised in the product of the first three PC vectors and their corresponding loading scores (Fig. 5). No significant differences were found in the use of the coordination patterns across visits or cadence conditions, nor in the EnHLs across visits. However, the EnHLs decreased with increased cadence. These findings support our prediction that a consistent neuromuscular control strategy is used during cycling by inexperienced cyclists.

### Muscle coordination is consistent across visit days and cadence conditions

A suboptimal coordination strategy during cycling can result in reduced power output and mechanical efficiency (Blake and Wakeling 2015). This observation highlights the importance of intermuscular coordination during pedalling. In our study, the PCA provided five to seven PCs from the sEMG dataset recorded across all conditions and visits. This finding is consistent with other studies demonstrating that six PCs explain ≥ 95% of the variance in sEMG datasets recorded from ten leg muscles during cycling by experienced cyclists (Wakeling and Horn, 2009; Hodson-Tole et al. 2020). In previous studies, the PCs have been considered to represent muscle synergies. If muscle activation is organised synergistically, then it is likely that muscle synergies would not vary during the same movement even if external factors such as cadence and visit day change. The robust consistency of the loading scores in this study indicate that, even in recreationally active individuals, the main coordination patterns are used to the same extent across cadence conditions and visits. This supports the idea that groups of muscles, rather than individual muscles are activated during cycling which may simplify motor demands (D’Avella et al. 2015) or regulation of low-level aspects of movement such as stresses within the joints (Alessandro et al. 2020).

Furthermore, our findings align with previous literature demonstrating that muscle synergies are consistent when cycling across different mechanical constraints (Hug et al. 2011). Previously, Hug and colleagues identified three synergies that accounted for the variance in sEMG data recorded from eleven leg muscles in trained cyclists. Similar to our findings, their synergy vectors showed robust consistency across a range of torque and velocity conditions. It is important to note however that in our study, a higher number of components was extracted than by Hug et al. (2011). This could be due to the training level of the cyclists involved or the decomposition algorithm used (Ting and Chvatal 2010). Nevertheless, the variations in dimensionality do not counteract the concept that a small set of muscle recruitment patterns contribute to motor coordination during cycling.

Moreover, this study demonstrated that the PC loading scores were repeatable across the two days, suggesting that consistent coordination patterns were used to the same extent on each day. This is important as muscle coordination patterns are evaluated pre- and post- cycling training interventions to identifying techniques which increase mechanical efficiency, power output and overall performance (Vigotsky et al. 2018). Longitudinal evaluations of muscle coordination are also used in clinical research to improve the outcomes of stroke survivors (Ambrosini et al. 2020), patients with neuromuscular diseases (Steele et al. 2015) and patients with motor injuries (Barroso et al. 2016). Thus, our findings reinforce the notion that such longitudinal measures are useful for clinical and sports researchers.

### PCA indicates personal coordination strategies

The reconstructed products of the PCs and their vector loading scores provide a visualisation of the correlated EMG intensities of individual muscles and their relative contribution to the overall EMG intensities. As such, we observed similar loadings for the quadricep muscles, the gastrocnemius, and the soleus, indicating a correlated activation of these muscles. Meanwhile, the semitendinosus, biceps femoris and tibialis anterior, showed opposite loading scores, suggesting that these muscles were antagonistically activated. The levels of antagonist muscle coactivation varied across the cyclists, likely indicating the techniques used and their varying levels of cycling experience (Candotti et al. 2009). Since recreationally active individuals were used, some participants may have been more experienced with meeting the task demands than others, for example through more frequent gym sessions or recreational cycling.

Nonetheless, the interindividual variations observed are not unique to these participants. Previous EMG studies have found large interindividual variations in the muscles activated within both groups of recreational cyclists (Jammes et al. 2001) and trained cyclists (Hug et al. 2008). Additionally, a recent study has shown that individuals can accurately be identified using a machine learning algorithm based on sEMG recordings of their lower limb muscles during pedalling (Hug et al. 2019). These findings suggest that individuals have unique muscle activation signatures which are used during cycling. This is important as it means that an individualised approach should be taken when observing muscle coordination parameters over time. The factors that determine these unique signatures are, however, unknown and warrant further investigation as they may improve understanding of how muscles are recruited for tasks of daily living.

### Entropic half-life reveals persistency in muscle coordination

The mechanical demands of cycling can alter the duration, timing, and amplitude of individual muscle activity (Blake and Wakeling 2015), that will contribute to features of different intermuscular coordination patterns. Fluctuations in the use of each coordination pattern can be examined by quantifying the short-term fluctuations of PC loading scores across consecutive pedal cycles (Hodson-Tole and Wakeling 2017). In this study, we found that the cycle-to-cycle variation in PC loading scores are not random but have a structure such that current pedal cycles influenced future cycles. There were large variations between participants in the number of pedal cycles over which signal structure persisted. Where fewer cycles are connected, it suggests each pedal cycle is treated more like an individual task and it is noticeable that EnHLs are skewed to lower values across the data set (Fig. 7). This may reflect a lack of familiarity with the task in these recreationally active individuals; however, cycle-to-cycle fluctuations in coordination have yet to be reported for more experienced cyclists so it is unknown what differences may occur with training.

While the EnHLs differed across the coordination patterns (different PCs), when the individual coordination patterns were compared the EnHLs were similar when cycling at each cadence across the visit days. This indicates that persistence in the cycle-to-cycle variation decayed over a similar number of pedal cycles for each coordination pattern when under the same conditions on each visit. We suggest that this consistency represents the repeated use of motor commands to activate muscle synergies. This finding aligns with the theory that muscle synergies are activated, rather than individual muscles to simplify motor redundancy problems (D’Avella et al. 2015) and/or reflect regulation of stresses within the joints (Alessandro et al. 2020) or other lower system-level requirements. Nonetheless, although no significant differences were found across visits, it is important to note that the effect sizes were small and with a larger sample size, significant differences may become more apparent. Thus, findings presented here should be considered in the context of these limitations, and further studies with a larger sample size are still required.

### Variability of entropic half-life across cadence conditions

Considering that muscle coordination varies across different mechanical demands of cycling (Blake and Wakeling 2015; Hodson-Tole et al. 2020), our findings of differences in EnHL between cadences (Fig. 7) indicate that the underlying control processes in recruitment are also influenced by cadence. We found a significant decrease in the persistence of the PC loading scores at 100 rpm compared to 60 rpm. This finding suggests that at the highest cadence, the individual pedal cycles had less influence on future pedal cycles. Shorter EnHLs indicate an increased number of solutions available to complete the task demands. Therefore, indicating that at the higher cadence there were more fluctuations in the muscle coordination possibly indicating participants searching for solutions to meet the task demand. It is possible that trained cyclists would show more connectivity across consecutive pedal cycles, using solutions they have learned to meet the task demand; however, this is an area that requires further investigation.

### Methodological considerations

The study population consisted of a small sample size of recreationally active individuals, who were untrained, so we could not investigate the effects of higher torque and velocity conditions. Nonetheless, the participants met the cadence and power output demands that were required by experimental design. Therefore, the repeatability of muscle coordination was still assessed in a variety of conditions, and the findings can be applicable to clinical patients who are likely not highly skilled cyclists. Furthermore, there were no significant differences in cadence across the visits and experimental conditions were tightly controlled to ensure that intersession repeatability was observed. Measurements were taken of electrode placement, and the height and position of the saddle and handlebars to minimize differences between visit days. Conditions were also presented in randomised order to minimise potential temperature and fatigue effects. The analysis and results presented are, however, dependent on PCs being calculated from physiologically relevant sEMG signals. It is important to remember that sEMG are open to non-physiological contamination from several sources, including time-varying voltage changes related to muscle motion and skin–electrode interface disturbance. While significant efforts were made to reduce contribution of noise (e.g. good skin preparation, wireless electrodes, discarding lower frequency signal content prior to calculation of intensity), noise could still have made contributions to the calculated intensities and the PCs should not be considered to purely reflect muscle coordination patterns. The careful preparation and analysis should, however, reduce their influence, leading us to interpret the PCs as being predominantly influenced by the muscle electrophysiology.

The number of pedal cycles analysed for the EnHL calculations were kept consistent to enable fair comparison across the conditions and visits. However, the EnHL values may have differed if a greater number of pedal cycles were analysed (Raffalt and Yentes 2018; 2020). It is also important to note that in this study, the EnHLs were analysed on a pedal-by-pedal basis. This approach has not been taken previously in studies where different cycling conditions are compared. The EnHL values cannot be directly compared with studies that have analysed the EnHLs across continuous EMG values and loading scores, as the timescales are very different (pedal cycles vs. milliseconds) although both analysis approaches suggest that fluctuations in muscle excitation are regulated from cycle to cycle (Pratt et al. 2021).

Finally, readers should note that this study was small, with a sample size of seven. This means that generalisability of the results to the wider population should be considered limited. Additionally, the sample was heterogenous, with both males and females asked to meet the same work demands (150 Watts). The differences in PCs quantified may have reflected differences in the relative work rate and responses of the individual. We have ensured that PCs were quantified for each individual participant and therefore comparisons across visits do reflect inter-individual consistency/alterations. The results are unique in assessing intermuscular coordination and its temporal organization in recreationally active individuals. Nevertheless, as stated previously, the limited sample does reduce the confidence with which findings can be generalized across the wider populations.

## Conclusion

This study investigated the intersession repeatability of the intermuscular coordination patterns used during cycling when pedalling at different cadences, across two visit days. A general consistency was indicated in the muscle recruitment patterns used across the visits and conditions. Additionally, the underlying structure of muscle coordination showed persistency which varied with cadence. Together these findings indicate that sEMG may be a reliable tool for observing changes in muscle coordination parameters over time. However, a large-scale study is required to confirm these findings and we suggest that an individualised approach should be taken when studying muscle coordination in cyclists.

## Data Availability

The data and custom-written code that support the findings of this study are available upon request from the corresponding author.

## Abbreviations

BF: Biceps femoris
EMG: Electromyography
EnHL: Entropic half-life
GL: Gastrocnemius lateralis
GM: Gastrocnemius medialis
GMax: Gluteus maximus
IQR: Interquartile range
PC: Principal component
PCA: Principal component analysis
RF: Rectus femoris
rANOVA: Repeated measures analysis of variance
SampEn: Sample entropy
sEMG: Surface electromyography
SD: Standard deviation
SOL: Soleus
ST: Semitendinosus
TA: Tibialis anterior
VL: Vastus lateralis
VM: Vastus medialis
